# Glutathione Involvement in Potato Response to French Marigold Volatile Organic Compounds

**DOI:** 10.3390/antiox13121565

**Published:** 2024-12-19

**Authors:** Jelena Savić, Đura Nakarada, Sofija Stupar, Ljiljana Tubić, Milica Milutinović, Miloš Mojović, Nina Devrnja

**Affiliations:** 1 Department for Plant Physiology, Institute for Biological Research “Siniša Stanković”—National Institute of the Republic of Serbia, University of Belgrade, 11108 Belgrade, Serbia; sofija.stupar@ibiss.bg.ac.rs (S.S.); tubic@ibiss.bg.ac.rs (L.T.); milica.milutinovic@ibiss.bg.ac.rs (M.M.); nina.devrnja@ibiss.bg.ac.rs (N.D.); 2 BioScope Labs, Center for Physical Chemistry of Biological Systems, Faculty of Physical Chemistry, University of Belgrade, 11158 Belgrade, Serbia; djura@ffh.bg.ac.rs (Đ.N.); milos@ffh.bg.ac.rs (M.M.)

**Keywords:** plant-to-plant communication, essential oil, priming defense, reactive oxygen species, antioxidative system

## Abstract

To elucidate the involvement of glutathione in the mitigation of induced oxidative changes and the sequestration of perceived volatiles in cells, we exposed potato plants to French marigold essential oil. The formation of short-lived radicals, the determination of scavenging activity towards ascorbyl and DPPH radicals, and the assessment of the potato plants’ overall intra/extracellular reduction status were performed using electron paramagnetic resonance spectroscopy (EPR). The results showed the presence of hydroxyl radicals in potatoes, with significantly reduced accumulation in exposed plants compared to the control group after 8 h. However, the kinetics of EPR signal intensity change for the pyrrolidine spin probe (3CP) in these plants showed very low reducing potential, suggesting that the antioxidant system acts lethargically and/or the probe has been reoxidized. Total glutathione and its reduced/oxidized form ratio, determined spectrophotometrically, showed that the exposed plants initially had lower glutathione levels with diminutive, reduced form compared to the control. Still, after 8 h, both characteristics were similar to those of the control. RT-qPCR analysis revealed that the volatiles altered the expression of glutathione metabolism-involved genes, especially that of glutathione-S-transferase, after 8 h. Glutathione metabolism was affected by volatiles in the initial response of potato plants exposed to French marigold essential oil, and glutathione molecules were involved in the mitigation of induced oxidative burst.

## 1. Introduction

In agroecosystems rich in diverse plant species, volatile organic compounds (VOCs) emitted by co-cultivating plants may contribute to the crops’ defense as natural inducers and defense priming effectors. These airborne signals could increase biotic/abiotic stress tolerance in neighboring plants [1] through cell wall reinforcement, enhanced antioxidant response, the induction of defense genes and/or the production of secondary metabolites [2]. Therefore, botanical diversity has been suggested as a sustainable strategy for reducing major threats in agriculture such as climate change, pest infestation and the negative footprint of overused chemicals [3]. This approach can be one of the proposed solutions for upgrading the green transition in agriculture through the revelation of naturally occurring mechanisms, processes and compounds that could increase the tolerance of plants to biotic and abiotic threats and ensure the sustainability of the desired agriculture transformation.

Plants sense the volatiles emitted by neighboring vegetation and function as VOC absorbents in the atmosphere. Flux field measurement over an orange plantation in California showed that almost 42% of the VOCs emitted in the plantation were deposited into surrounding vegetation [4]. A portion of the emitted VOCs can be attached to the surface of neighboring plants or penetrate and be partitioned into receiving plant tissues [5]. Interestingly, Capone et al. [6] reported that the concentration of the Eucalyptus fragrance component 1,8-cineole, commonly known as eucalyptol, in red wines is directly influenced by the proximity of eucalyptus trees to grapevines grown nearby. In two distinct vineyards, the quantity of 1,8-cineole in the wines made from those grapes decreased with the distance between the grapevines and the trees.

Although the mechanisms of perception of VOCs by plants are poorly understood, it is known that a part of attached VOCs is taken up by the receiving plant tissues through a physicochemical process [7]. The first event in the plant response to the perception of VOCs is a change in the plasma membrane potential of the exposed plant cells [8,9] and an accompanying change in ion fluxes across the membrane, especially of calcium (Ca^2+^) [10]. In addition, the downstream effect of the diffusion of VOCs into the cytosol implies glycosylation and glutathionylation processes involved in VOC metabolizing, leading to a further increase in VOC uptake [11]. The import of Ca^2+^ into the cytosol as a result of VOC diffusion is a signal that can trigger cellular responses involving the production of reactive oxygen species (ROS) and the activation of signal transduction processes, which subsequently regulate vital functions in plants [12]. The induced oxidative burst immediately triggers both components of the antioxidant machinery, the non-enzymatic and the enzymatic, which aims to prevent oxidative stress-induced damage to the vital plant molecules (proteins, nucleic acids). These triggered responses are precisely the starting point from which a later component of the plant response to VOCs could be regulated and defense primed via the phytohormone network and associated gene expression. Interestingly, evidence for the involvement of VOC stimuli and subsequent signaling in plant memory has accumulated in recent years. The memory of previous exposure to VOCs can “prime” plants and prepare their responses to upcoming stresses, triggering faster, more robust and energy-efficient responses [13,14,15].

Together with ascorbate, glutathione is a core component of the non-enzymatic redox hub that regulates ROS levels in plants by acting as ubiquitous ROS scavengers (H_2_O_2_, ^•^OH, O_2_^•−^, ^1^O_2_) [16]. This tripeptide, which consists of glycine, cysteine and glutamic acid, can occur in free form or bound to other molecules. Free forms of glutathione occur in the cell in two reversible states: reduced and oxidized. Under normal, non-stressful conditions, the reduced form of glutathione (GSH) predominates over the oxidized form (GSSG), and it is their ratio that is an indicator of the stress state of the cell [17,18]. The initial reaction of the plant to an increased ROS content caused by unfavorable environmental conditions, suboptimal growth conditions, xenobiotic compounds (e.g., VOCs) and/or stress, is the enhancement of antioxidant defense which manifests itself in an increase in GSH as a non-enzymatic antioxidant, together with other partner compounds such as ascorbic acid, phenols and secondary metabolites. Subsequently, the enzymatic components of antioxidant defense, such as superoxide dismutases (SODs), catalases (CATs), peroxidases (PODs) and ascorbate peroxidase (APX), are included. In addition, antioxidant enzymes, such as monodehydroascorbate reductase (MDHAR), dehydroascorbate reductase (DHAR), glutathione reductase (GR), glutathione S-transferase (GST) and glutathione peroxidase (GPX), are involved in these stress responses. There is accumulated evidence that environmental conditions regulate the transcriptional level of gene encoding enzymes involved in GSH biosynthesis; however, this process is additionally regulated at the post-translational level by a GSH-GSSG redox cycle [19,20]. Multiple levels of regulation ensure rapid and efficient responses to stress situations. Gamma-glutamyl cysteine synthetase (ƴ-GCS) and glutamate-glutathione synthetase (GSS) are enzymes that catalyze the biosynthesis of GSH via two ATP-dependent steps. The third enzyme, gamma-glutamyltranspeptidase 3 (GGT), plays a vital role in the gamma–glutamyl cycle by catalyzing the direct degradation of GSH and providing L-glutamate as a precursor for the new cycle of GSH biosynthesis. Under stress, GSH is oxidized to GSSG in the presence of glutathione peroxidase (GPX), while NADPH-dependent glutathione reductase (GR) is required for regeneration to GSH, but this enzyme also mediates the ascorbate–glutathione cycle. Importantly, APX/DHAR and GR also link the redox potential of ascorbate and the NADPH/NADP+ potential to the GSH redox potential [21].

On the other hand, the fate of perceived VOCs can go in several directions after penetration and after triggering the plant defenses. Plants must alter these chemicals for long-term storage as non-toxic compounds because they are unable to excrete waste products. Apart from the possibility of being incorporated into primary metabolism or phytohormone biosynthesis [22,23], perceived VOCs may undergo xenobiotic detoxification mechanisms such as glycosylation and glutathionylation [7]. While glycosylation primarily prevents self-intoxication by sequestering endogenous VOCs into a cellular compartment [24], glutathionylation is an important pathway for the detoxification of exogenous xenobiotic VOCs through the formation of conjugates with GSH [25]. A superfamily of multifunctional glutathione-S-transferase (GST) enzymes plays the major role in glutathionylation, but the conjugates can also be formed non-enzymatically with compounds that exhibit reactive electrophilicity. The sulfhydryl group (-SH) on the cysteine residue of the GSH molecule is the reactive component required for this reaction. By eliminating gamma-glutamyl from the extracellular GSH, the enzyme gamma-glutamyl transpeptidase (GGT) breaks it down into cysteinyl-glycine or cysteinyl-glycine conjugates, which are further degraded by dipeptidases [26]. In tomato plants exposed to methacrolein at lower doses, the formation of GSH-adduct was the main metabolic process in the tissue [27]. Once the available GSH in the plant cells was depleted, the efficiency of methacrolein absorption decreased dramatically under high-dose exposure conditions, suggesting that the formation of GSH-adduct was the driving force in methacrolein uptake. Transporters that recognize the GSH conjugated to a xenobiotic have been identified in the tonoplast membranes of plants [28].

The overall aim of this study was to investigate the potential of VOCs to affect the physiology of receiving plants and induce stress-mitigating mechanisms, using the companion plants potato (*Solanum tuberosum* L.) and French marigold (L.), which are commonly grown together in traditional and organic agriculture. We investigated the initial circumstances that occur in potato when exposed to French marigold essential oil (FM-EO), i.e., the production of short-lived radical species in potato leaves with an additional assessment of the total intra/extracellular redox status. To reveal the antioxidant mechanisms used to attenuate FM-EO-induced changes in the potato plants, glutathione metabolism was investigated and the total glutathione content, and the ratio of its reduced and oxidized forms, were determined. The involvement of glutathione in coping with the consequences of FM-EO perception in potato plants was supported by the results of de novo bioinformatic annotation of cDNA microarray data of potato reported by Stupar et al. [29], so an additional analysis of the expression levels of nine genes involved in overall glutathione metabolism (biosynthesis, redox cycle and glutathione-involved VOC cellular sequestration) was performed. All responses were monitored in potato plants exposed to FM-EO for different time periods (2, 4, 6 and 8 h). Moreover, the potential of FM-EO to induce the plants into a ‘primed’ state in which defense is elevated for some time after VOC stimulus was tested in potato exposed to FM-EO over 3 consecutive days for 8 h each day, and samples were collected 5 or 10 days after EO removal.

## 2. Materials and Methods

### 2.1. Experimental Design

The potato plants (*Solanum tuberosum* L. cultivar Désirée) used in the experiments were germinated in the lab from tubers collected from a chemically untreated private field (Sremska Mitrovica, Serbia, 44°58′12″ N 19°36′45″ E). The plant material of French marigold (*Tagetes patula* L.) used for EO isolation was collected at the blooming stage from a private garden near Belgrade (Jajinci village, Serbia, 44°44′ N 20°29′ E). Seeds were purchased from Semenarna Ljubljana (Ljubljana, Slovenia, cat no. 67670).

**Figure 1:**
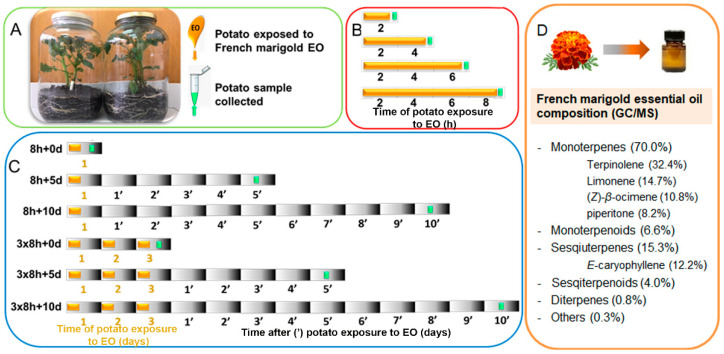
Experimental setup. (**A**) Young soil-grown potato plants were exposed to French marigold essential oil (EO) for different time periods in tightly closed glass jars, while controls without EO were maintained under the same conditions. (**B**) Potato material was collected immediately after single exposure to EO for 2, 4, 6 and 8 h in order to examine the effect of EO on antioxidant scavenging activity and glutathione metabolism. (**C**) For the analyses of French marigold EO’s priming effect on the expression of genes related to glutathione metabolism, material was collected immediately (0 d), 5 days (5 d) and 10 days (10 d) after single exposure for 8 h (8 h) or three-times-repeated 8 h long (3 × 8 h) EO exposure treatment. (**D**) The phytochemical composition (%) of French marigold essential oil was used in this study. The complete list of compounds has previously been published [29].

Potato growth conditions, French marigold essential oil (FM-EO) extraction and chemical composition were previously described by Stupar et al. [29]. Briefly, young well-developed potato plants were grown individually in 5 L glass jars on soil mixture. The EO was applied to the filter paper (10 µL) and placed in the jar on the metal holder, ensuring no direct contact with the potato plants, and then the jars were tightly closed with lids and sealed with parafilm. Jars containing the control plants were maintained under the same conditions, but without exposure to EO (Figure 1A). Potato plants were exposed either to single EO treatment for different time periods (2, 4, 6 and 8 h) in order to examine the induction of potato responses to EO (Figure 1B), or to single (8 h) or three-times-repeated (during three consecutive days) 8 h long (3 × 8 h) EO treatment in order to examine the priming effect of EO exposure immediately (0 d), or after 5 or 10 days (5 and 10 days, respectively; Figure 1C). Plant material (potato leaves) was collected from 3 or 4 plants (biological replicates) per treatment, homogenized in liquid nitrogen, and further processed for different analyses. To determine the production of short-lived radical species and evaluate the overall redox status, intact potato leaves were used.

### 2.2. Examination of Short-Lived Radicals’ Production and Antioxidant Scavenging Activity of Potato Plants Exposed to French Marigold EO

For the determination of radicals’ production and turnover in potato tissues upon receiving the French marigold VOCs, electron paramagnetic resonance (EPR) spectroscopy was employed. All EPR spectra were recorded using a Bruker ELEXSYS-II X-band spectrometer (Rheinstetten, Germany) equipped with an R4123SHQE X-band resonator. EPR spectra were analyzed using Xepr software (ver. 2.6b.84; Bruker BioSpin, Ettlingen, Germany). The quantification of EPR signals was performed by calculating the area under the spectra, which directly correlates with the concentration of the analyzed radicals. Hydrogen peroxide, MeOH (methanol; MS grade), DPPH (2,2-Diphenyl-1-picrylhydrazyl), 3CP (3-Carbamoyl-2,2,5,5-tetramethyl-3-pyrrolidin-1-oxyl) and 3CxP (3-Carboxy-2,2,5,5-tetramethyl-3-pyrrolidin-1-oxyl) were purchased from Sigma-Aldrich (Steinheim, Germany). Spin trap DEPMPO (5-(Diethoxyphosphoryl)-5-methyl-1-pyrroline-N-oxide) was purchased from Focus Biomolecules (Plymouth Meeting, PA, USA). AccuGENE deionized 18 MΩ water was purchased from Lonza (Bornem, Belgium). Iron (II) sulfate heptahydrate, iron (III) chloride, EDTA (ethylenediaminetetraacetic acid tetrasodium salt), ascorbic acid and DMSO (dimethyl sulfoxide) were purchased from Merck (Darmstadt, Germany). All chemicals were of analytical grade.

#### 2.2.1. Determination of Short-Lived Radicals’ Production in Potato Leaves

**Figure 2:**
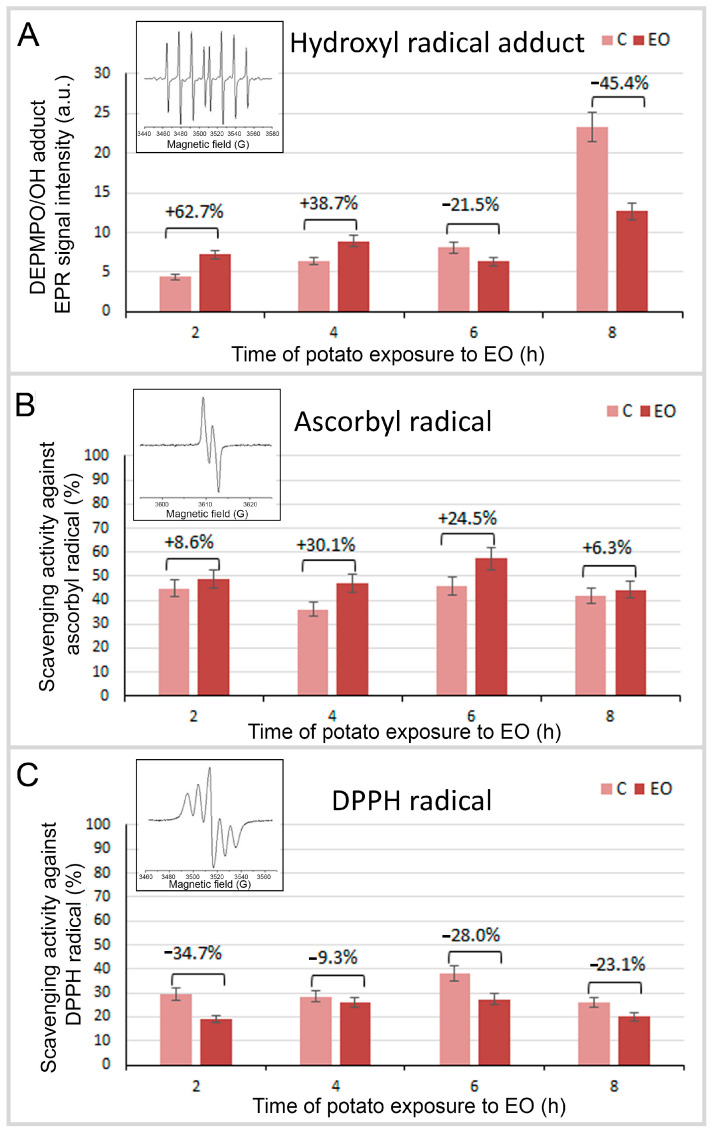
Short-lived radical production and assessed antioxidant scavenging activity of potato plants exposed to French marigold EO. (**A**) EPR signal intensities of DEPMPO/OH adducts corresponding to the amount of hydroxyl radical (^•^OH) trapped upon their production in the potato leaves. Small amounts of carbon-centered and hydrogen radicals were also detected (inserted spectrum). Scavenging activity (%) of potato leaf MeOH extracts towards (**B**) ascorbyl radicals (^•^Asc) and (**C**) DPPH radicals (representative EPR spectra of ^•^Asc and DPPH radicals are presented as inserts). The DEPMPO adduct EPR signal intensities were measured from intact leaves, and the radical scavenging activities were measured from leaf extracts obtained from potato plants exposed to French marigold essential oil (EO) for 2, 4, 6 or 8 h, or in unexposed control plants (**C**). EPR signal intensities were quantified by measuring the area under the spectra. Values are the means of three biological replicates (n = 3).

To compare the accumulation of short-lived radicals in FM-EO-exposed and control potato leaves, the spin trap DEPMPO was utilized [30,31]. This spin trap was chosen for its well-established selectivity and the extended half-life of spin adducts [32]. In a concise procedure, following exposure to the FM-EO for the specified duration (2, 4, 6 and 8 h), a section of the leaf (approximately 20 mg) was positioned on the tissue cell and covered with 5 µL of DEPMPO (100 mM). The leaf section was incubated with the spin trap for 5 min, after which EPR spectra were recorded in the X-band (Figure 2A). Recordings were made using the following experimental settings: a microwave power of 10 mW, a microwave frequency of 9.85 GHz, a modulation frequency of 100 kHz and a modulation amplitude of 1 G.

#### 2.2.2. Determination of the Scavenging Activity of Potato Leaf Extracts Towards Ascorbyl (^•^Asc) Radicals

For the analyses of potato scavenging activity against ascorbyl (^•^Asc) and DPPH radicals, extracts of potato leaves exposed to FM-EO were made by the extraction of 100 mg of a liquid nitrogen-frozen, grounded material in 1 mL MeOH during the night and filtered through Whatman No 3. For all analyses, the potato plants were exposed to FM-EO for 2, 4, 6 and 8 h, and each time point had a corresponding control.

To detect the activity of potato leaf MeOH extracts towards ascorbyl radicals, the EPR signal of ^•^Asc in DMSO solution was recorded in the presence of extracts using the procedure based on a previously developed method [33]. In brief, 10 µL EDTA (final concentration 250 µM) and 1 µL iron (III) chloride (final concentration 8 µM) were mixed to form the Fe(III)-EDTA complex in 74 µL of DMSO, and the ^•^Asc radical was generated upon the addition of 10 µL of ascorbic acid (final concentration 250 µM) into the system. At this point, 5 µL of potato leaf extract in MeOH was added, and 30 µL of this mixture was transferred into the gas-permeable Teflon tube. The X-band EPR spectra of ^•^Asc radical were recorded after 2 min, using the following experimental settings: microwave power of 10 mW, microwave frequency of 9.85 GHz, modulation frequency of 100 kHz, and modulation amplitude of 2 G (Figure 2B). Control recordings were made by substituting the samples with the same volume of the solvent. The antiradical activity of the extracts (AA) is expressed as follows:

AA = (Ic − Ia)/Ic × 100 (%),(1)

where Ic and Ia refer to the double integral values of the ^•^Asc signal in recording control and extracts, determined from the EPR spectra, respectively.

#### 2.2.3. Determination of the Scavenging Activity of Potato Extracts Towards DPPH Radicals

The distinct shape of the DPPH EPR spectrum facilitates the determination of the initial radical concentration and the efficacy of potato leaf MeOH extracts in reducing their presence in the system. The DPPH reduction activity of extracts derived from potato leaves was investigated employing the previously outlined EPR methodology [34,35]. For each reaction mixture, 1 µL of extract was introduced to 29 µL of DPPH solution in 80% MeOH (final concentration 210 µM). The resultant mixture was then transferred into a gas-permeable Teflon tube, and the X-band EPR signal was recorded after 2 min, utilizing the specified experimental settings: a microwave power of 10 mW, a microwave frequency of 9.85 GHz, a modulation frequency of 100 kHz and a modulation amplitude of 2 G (Figure 2C). Control recordings were made by substituting the samples with the same volume of the solvent. The antiradical activity of the extracts (AA) is expressed using the same formula as in Section 2.2.2 Equation (1).

### 2.3. Estimation of the Capacity of Potato Leaf Tissues to Alter the EPR Signal of Pyrrolidine Spin Probes (X-Band 1D Gradient Imaging)

**Figure 3:**
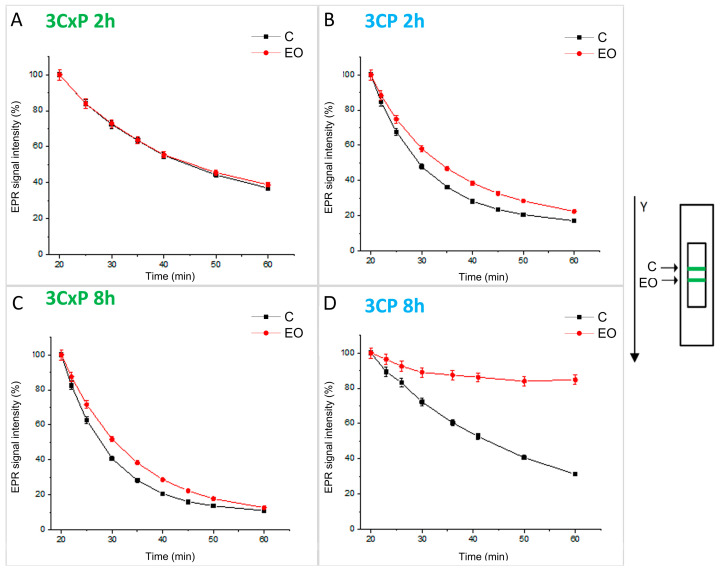
The capacity of potato leaf tissues to alter the EPR signal of pyrrolidine spin probes. Kinetics of change in the EPR signal intensity of spin probes 3CxP and 3CP in strips of potato leaf after 2 h ((**A**) and (**B**), respectively) and 8 h ((**C**) and (**D**), respectively) of treatment with EO (red circles) and the corresponding controls (black squares). The arrangement of leaf strips from control (C) and EO exposed (EO) plants on tissue cell, along with the direction of 1D EPR imaging, is illustrated on the right side. Values are the means of three biological replicates (n = 3).

To assess the intra/extracellular total redox status of potato leaf samples, pyrrolidine spin probes, membrane-permeable (3CP) and membrane-impermeable (3CxP) [36], were employed. Subsequent to the treatment with FM-EO, leaf strips measuring 5 × 2 mm were excised and incubated for 5 min under vacuum conditions with the spin probes (1 mM 3CP and 5 mM 3CxP, respectively). The identical procedure was followed for unexposed potato leaves, serving as the control. The cutouts were placed onto a quartz tissue cell, one above the other, and recorded simultaneously using an X-band 1D gradient EPR experiment along the *Y*-axis (Figure 3). The recording parameters were as follows: microwave power of 10 mW, microwave frequency of 9.8 GHz, modulation frequency of 100 kHz, modulation amplitude of 2 G, and magnetic field gradient of 20 G cm^−1^. The applied gradient value was optimized to achieve the optimal separation of nitroxide EPR peak signals from each tissue sample. The double integral values of the EPR signal for each leaf strip were measured over a 60 min period.

### 2.4. Spatiotemporal Visualization of the Capacity of the Potato Leaf Tissues to Alter the EPR Signal of Pyrrolidine Spin Probe (X-Band 2D Imaging)

**Figure 4:**
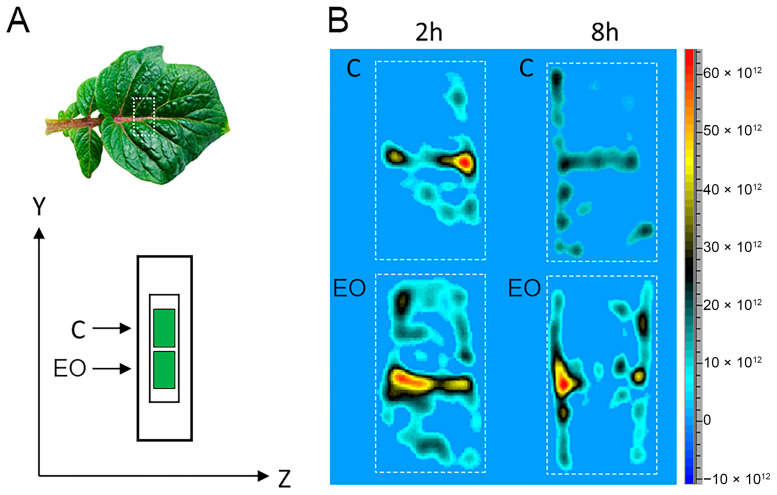
Spatiotemporal visualization of the potato leaf tissues’ capacity to alter the EPR signal of a pyrrolidine spin probe. Typical potato leaf with highlighted section used for the 2D EPR imaging experiment ((**A**), upper panel). Schematic representation of control (C) and treated (T) leaf samples, positioned in the ZY-plane within the EPR tissue cell ((**A**), lower panel). Two-dimensional EPR images of control (C) and treated (EO) potato leaf samples after 2 or 8 h of treatment with FM-EO, followed by incubation in the spin probe 3CP (**B**).

To visualize the intra/extracellular total redox status of potato leaves, a membrane-permeable pyrrolidine spin probe (3CP) was used. For this purpose, 5 × 7 mm samples excised from potato leaves were soaked in 67 mM 3CP for 15 min under vacuum, placed onto a quartz tissue cell, and recorded using an imaging experiment in the ZY-plane (Figure 4) utilizing the following parameters: a microwave power of 10 mW, a microwave frequency of 9.8 GHz, a modulation frequency of 100 kHz, a modulation amplitude of 2 G and a magnetic field gradient of 20 G cm^−1^.

### 2.5. Determination of Glutathione Content in Potato Plants Exposed to French Marigold Essential Oil

The glutathione content was determined according to Horváth et al. [37] and Sahoo et al. [38] with some modifications. Potato leaf tissue (200 mg) was rapidly powdered in liquid nitrogen. Subsequently, 800 µL of 5% methaphosphoric acid was added. The homogenate was centrifuged at 12,000× *g* for 20 min at 4 °C, and the supernatant was collected for determination.

The total glutathione concentration was measured directly in the supernatant using an enzymatic assay. The oxidized glutathione (GSSG) content was determined in the extract, which was supplemented by 2-vinylpyridine to mask reduced glutathione (GSH). The first reaction mixture consisted of 0.1 M potassium phosphate buffer (pH 7.5), 10 mM 5,5-dithio-bis-(2-nitrobenzoic acid) (DTNB; Sigma-Aldrich, St. Louis, MO, USA), and 20 µL tissue extract in 950 µL total reaction mixture. After 5 min of incubation at room temperature, the absorbance was read at 405 nm. The mixture was then returned to the tube and 4.80 mM NADPH (Sigma-Aldrich) and 1 U glutathione reductase (GR from baker’s yeast) were added. The incubation was then continued at room temperature for 25 min. Standard curves were obtained for GSH and GSSG in the range of 0–100 µM and 0–2.5 µM, respectively.

### 2.6. Analysis of French Marigold Essential Oil Effects on Expression of Genes Involved in Glutathione Metabolism

#### 2.6.1. In Silico Annotation of cDNA Microarray Data with Glutathione Metabolism Pathway

RNA isolated from samples of potato exposed for 8 h to FM-EO (Figure 1B) and corresponding control plants were analyzed by complementary DNA (cDNA) microarray, and the results were reported by Stupar et al. [29]. The expression of genes involved in glutathione metabolism was extracted from microarray data processed in R 3.6 [39], which was for the purposes of this study subjected to de novo bioinformatic analyses which included cross-referencing microarray data with the KEGG Pathways Database (Kyoto Encyclopedia of Genes and Genomes) for the glutathione metabolism pathway (sot00480). The results shown correspond to the fold change (FC) values (with FC ≥ 2 cut off) of differentially expressed transcripts (*p* ≤ 0.05, n = 4). The microarray results for glutathione-related genes are available at the trusted digital repository RADaR, at http://radar.ibiss.bg.ac.rs/handle/123456789/7014 (all accessed on 26 September 2024).

#### 2.6.2. RT-qPCR Analysis of Expression of Genes Involved in Glutathione Metabolism

To confirm the microarray results and study the temporal expression patterns of selected genes related to glutathione metabolism in potato affected by FM-EO, RT-qPCR was employed. Samples for analyses were collected from potato plants exposed to FM-EO for different time periods (2, 4, 6 and 8 h) (Figure 1B), or from plants exposed to single (8 h) or three-times-repeated (during three consecutive days) 8 h long (3 × 8 h) EO treatment in order to examine the priming effect of EO exposure immediately after EO exposure termination (0 d), or after 5 or 10 days (5 d and 10 d, respectively; Figure 1C). The total RNA isolated in biological triplicates (n = 3) from EO-receiving potato leaves [29] was subjected to reverse transcription (RT) using the GeneAmp™ RNA PCR Core Kit (Applied Biosystems™, Waltham, MA, USA cat. no. N8080143) with oligo d(T)16 primers, according to the manufacturer’s instructions. Real-time qPCR was performed on the Applied Biosystems QuantStudio 3 Real-Time PCR System using SYBR Green (Maxima SYBR Green/ROX qPCR Master Mix (2×), cat. no. K0221; Thermo Scientific, Waltham, MA, USA). The reaction mixture contained 1 µL of cDNA corresponding to 20 ng RNA and primers for selected genes involved in potato glutathione metabolism (*ƴ-GCS*, *GSS*, *GGT*, *GR*, *GPX*, *IDH*, *GSTL3*, *GSTT1*, *GSTa*; Supplementary Table S1), designed using Primer-BLAST (www.ncbi.nlm.nih.gov/tools/primer-blast). The specificity of all the primers was validated by BLAST, the separation of RT-PCR products on 1.2% agarose gels, and the analysis of melting curves during qPCR amplification. The results were analyzed using QuantStudio™ Design and Analysis Software version 1.4 (Thermo Fisher Scientific, Waltham, MA USA). The expression levels of analyzed genes were normalized to the internal control, 18S rRNA, and calculated relative to each non-exposed control according to the 2^−ΔΔCt^ method [40]. The results were presented as the log_2_ transformation of fold changes (log_2_FC). For each gene at each time point, a Student’s *t*-test was used for comparison between treatment and corresponding control, and statistically different means are denoted with one asterisk (*) for *p* ≤ 0.05 or with two asterisks (**) for *p* ≤ 0.10. The means of different treatments (2, 4, 6 and 8 h) were each compared with Fisher’s least significant difference (LSD) test at *p* ≤ 0.05, and different letters denote statistically different means (n = 3).

## 3. Results

### 3.1. Production of Short-Lived Radical Species by Potato Leaves Exposed to French Marigold EO

Upon adding the spin-trap DEPMPO to intact leaf cutouts, the predominant signal observed was of the DEPMPO/OH adduct, indicating a dominant production of ^•^OH radicals (Figure 2A). Only a small amount of other adducts, originating from carbon-centered and hydrogen radicals, were detected (see Figure 2A—inserted spectrum). The production of ^•^OH in control leaves (grown in closed glass jars without EO) gradually increased over time, peaking after 8 h. A similar increase was observed in FM-EO-exposed plants during the first 4 h, with a higher accumulation of ^•^OH radicals compared to the controls. However, EO exposure led to a decrease in ^•^OH accumulation after 6 and 8 h compared to the control samples. Specifically, a notable reduction of 21.5% in the DEPMPO/OH signal was observed after 6 h, which increased to 45.4% after 8 h. This indicates an EO-induced decrease in the concentration of these biologically relevant radicals in the exposed potato plants.

### 3.2. Radical Scavenging Activity of Potato Exposed to French Marigold EO

The measurement of the potential of potato leaf MeOH extracts to scavenge ^•^Asc radicals revealed that all EO-exposed potato plants displayed increased scavenging activity compared to their controls (Figure 2B). The greatest increase, at 30.1%, was observed after 4 h of exposure, while the highest overall scavenging activity was recorded after 6 h, for both exposed and control plants. This result is particularly significant given the biological importance of ascorbyl radicals.

The opposite trends were observed in the case of scavenging activity toward DPPH radicals (Figure 2C). Based on these findings, it can be inferred that extracts from unexposed control potato plants consistently exhibited higher scavenging activity compared to their EO-exposed counterparts, with the highest reduction of 34.7% detected in the shortest EO exposure treatment (2 h). Similar reduction compared to corresponding control was seen after 6 and 8 h long EO exposure (28.0% and 23.1%, respectively). Over the course of the experiment, there were no notable variations in DPPH scavenging activity between short-term (2 h) and extended exposure durations (8 h), across both control and exposed groups. The peak values were noted at 6 h, but the overall scavenging activity was lower against DPPH than ^•^Asc radicals.

### 3.3. Capacity of Potato Leaf Tissues to Alter the EPR Signal of Pyrrolidine Spin Probes

A kinetic study of pyrrolidine spin probe EPR signal change was conducted for short (2 h, Figure 3A,B) and extended (8 h, Figure 3C,D) exposures to FM-EO. The acquired results showed that leaves exposed to FM-EO (red circles) exhibited comparatively similar redox activity towards both 3CP and 3CxP spin probes as unexposed controls (black squares), with the only major exception observed for intra/extracellular spin probe 3CP with leaves which undergo 8 h long EO treatment (Figure 3D). In the case of 3CP, even 2 h of EO treatment affected slower redox activity for treated samples (Figure 3B). A similar effect was also observed for extracellular spin probe 3CxP, but only after 8 h of treatment. However, for the spin probe 3CP, 8 h of EO treatment significantly amplified the difference between EO and control samples, primarily because the EO-exposed plant did not exhibit any major change in the 3CP redox state, reaching a plateau after 30 min.

Figure 4 illustrates the experimental set-up (Figure 4A), marking the location of the excised leaf section and the sample’s position in the resonator during the performance of the 2D imaging experiment, which is intended to illustrate the spatiotemporal distribution of the spin probe 3CP. Analysis of the image reveals that the central vein of the leaf exhibited the highest absorption of the spin probe, with a significantly lower signal detected in the surrounding tissue (Figure 4B). Although it remains unclear whether the reduced signal intensity in the surrounding tissue indicates lower spin probe absorption or increased redox activity, it was observed that both short (2 h) and prolonged (8 h) exposure of leaves to FM-EO generally resulted in higher 3CP signals compared to untreated samples.

### 3.4. Total Glutathione Content and GSH/GSSG Ratio

**Figure 5:**
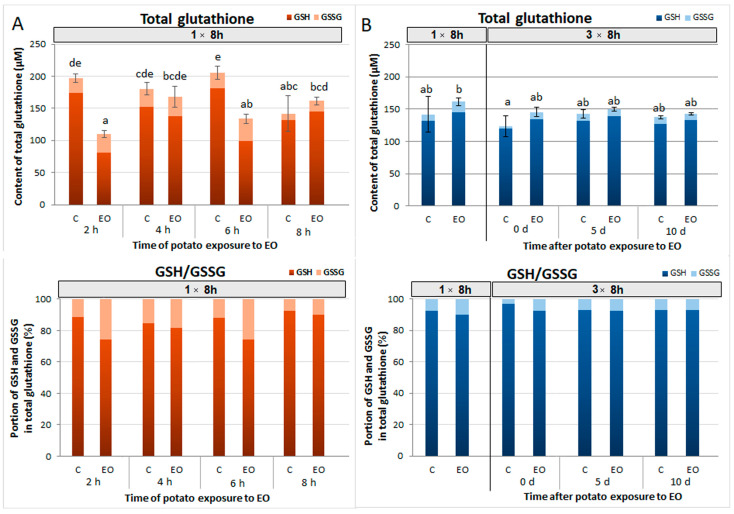
Glutathione content in potato plants exposed to French marigold essential oil. Total glutathione content with share of reduced GSH and oxidized GSSG forms and percentage portion of GSH and GSSG in total glutathione, in (**A**) potato plants exposed to French marigold essential oil (EO) for 2, 4, 6 and 8 h, and corresponding controls (C), and in (**B**) plants after exposure to French marigold EO for single (1 × 8 h) or three-times-repeated 8 h long (3 × 8 h) periods immediately (0 days) or 5 and 10 days after repeated exposure. The results are presented as means ± standard errors (n = 3) and separated by Fisher’s least significant difference (LSD) test. Bars with different letters denoted statistically different values at *p* ≤ 0.05.

For the concentration of total glutathione, its reduced (GSH) and oxidized (GSSG) forms were measured spectrophotometrically to obtain insight into the redox state of potato plants exposed to FM-EO. The results showed that the concentration of total glutathione after single exposure, when compared to the corresponding controls, decreased significantly in potato plants after initial exposure (2 h) to FM-EO (Figure 5A). During prolonged exposure (4 and 6 h) the content of glutathione slightly fluctuated under the influence of FM-EO, reaching the levels determined in control plants after 8 h. In all samples, the reduced GSH form was dominant over the oxidized GSSG form (Figure 5A). After initial 2 h-long exposure to FM-EO, a share of oxidized GSSG was increased in total glutathione content from 11.6 to 26%. Over next 4 h (4 and 6 h of exposure) the presence of FM-EO increased the level of GSSG compared to the corresponding controls. However, at prolonged EO exposure (8 h), the level of GSSG turned to the control level with a noteworthy dominance of the reduced form, with 7.3 and 10.1% in the control and EO-exposed potato, respectively.

Repeated exposure of potato plants to FM-EO (3 × 8 h) did not significantly affect total glutathione content nor the portion of its reduced and oxidazed forms (Figure 5B) compared to single exposure (1 × 8 h). Consequently, during the next 10 days, this tretament (3 × 8 h) did not provoke any change in glutathione accumulation, maintaining the levels of total glutathione and GSH/GSSG portions at the level of the controls.

### 3.5. Expression of Genes Involved in Glutathione Metabolism Pathway in Potato Plants Exposed to French Marigold EO

Biostatistical analysis of cDNA microarray-obtained data revealed that from 124 sequences annotated in the glutathione metabolism pathway in potato (sot00480), 43 had a significantly altered expression (35%), and 42 were upregulated after 8 h long exposure to FM-EO (http://radar.ibiss.bg.ac.rs/handle/123456789/7014).

**Figure 6:**
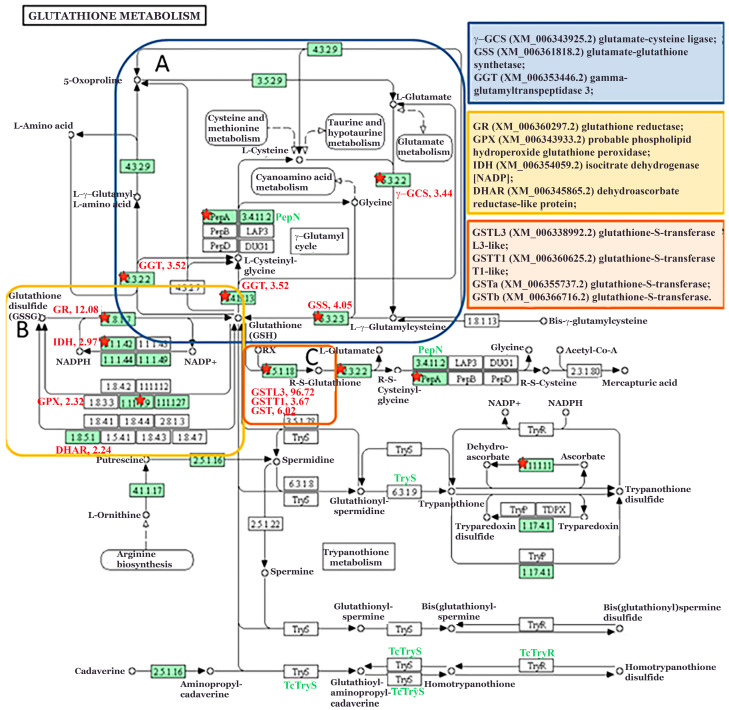
Dynamic visualization of gene expression onto KEGG glutathione metabolism pathway diagram for potato (sot00480). Map shows the different proteins involved in (**A**) glutathione biosynthesis, (**B**) GSH-GSSG redox cycling and (**C**) *GST*s involved glutathionylation. Green squares are hyperlinked to genes in the potato reference pathway. Genes with statistically significant fold change (FC ≥ 2, *p* ≤ 0.05) are highlighted with red asterisks, and gene abbreviation and value for FC are written next to the square.

After aligning these sequences with the KEGG pathway, 13 different genes with significantly increased expression (FC ≥ 2 and *p* ≤ 0.05) were identified and presented on the pathway diagram (Figure 6). Three of them belong to the GSH biosynthesis branch (Figure 5A), converting L-glutamate to GSH directly through two consecutive steps coded by *γ-GCS* (glutamate-cysteine ligase) and *GSS* (glutamate-glutathione synthase), both with an increased FC of 3.44 and 4.05, respectively. The third enzyme, *GGT* (gamma-glutamyltranspeptidase 3), providing L-glutamate as a precursor for the new cycle of GSH biosynthesis, was also increased with an FC of 3.52.

The strong induction was also seen in genes involved in the GSH-GSSG redox cycle (Figure 6B), with the FC of the *GPX* and *GR* genes being 2.32 and 12.08, respectively. A similar induction (FC = 2.97) was measured also for *IDH*, which is responsible for turning the oxidized form of NADP+ cofactor into its reduced form, NADPH, involved in the regeneration of GSH. The third part of the GSH metabolism pathway, the glutathionylation process of GSH conjugation catalyzed by numerous *GSTs* (Figure 6C), was the most affected by EO exposure during 8 h, according to significant induction in expression of some transferases, with *GSTL3* having an FC over 96.

**Figure 7:**
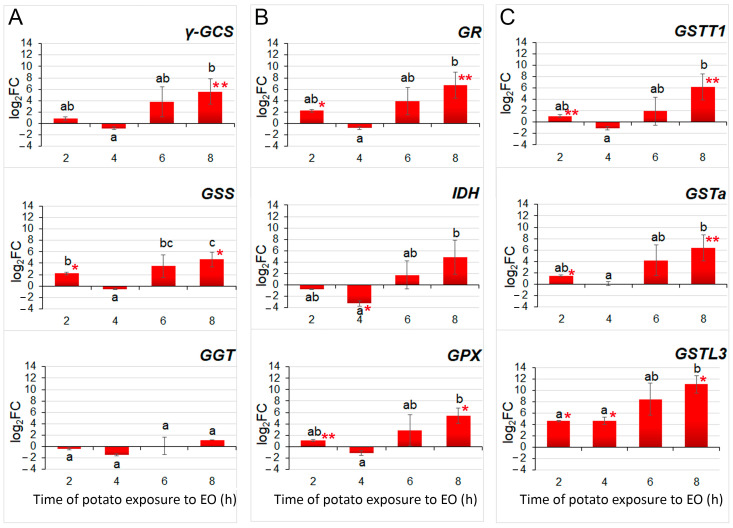
RT-qPCR-obtained expression profiles of potato genes involved in the GSH metabolism. Expression of genes involved in (**A**) biosynthesis of GSH, (**B**) redox conversion of GSH and (**C**) conjugating GSTs after exposure to French marigold EO for different time periods (4, 6, 8 and 12 h). The fold change (FC) of the gene’s expression was obtained after ΔΔCt normalization to the expression of reference 18S gene and to the expression in unexposed controls (log_2_FC = 0) for each time point, and presented after log2 transformation (log_2_FC). Means of three biological replicates (n = 3) were compared with one-way ANOVA and presented with standard errors. Letters above the bars denote significant differences according to Fisher’s LSD post-hoc test at *p* ≤ 0.05. For each gene at each time point, Student’s *t*-test was used for comparison between treatment and corresponding control, and statistically different means are denoted with one asterisk (*) for *p* ≤ 0.05 or with two asterisks (**) for *p* ≤ 0.10.

To study the expression pattern of identified GSH metabolism-related genes in more detail, RT-qPCR was employed on samples collected from the potato plants exposed to single FM-EO treatment lasting for 2, 4, 6 and 8 h (Figure 1B and Figure 7). The results highlighted very similar expression patterns for all nine analyzed genes belonging to GSH biosynthesis (Figure 7A), the GSH-GSSG redox cycle (Figure 7B), and glutathionylation (Figure 7C). For all, gene expression increased with prolonged exposure, with the maximal induction being reached after 8 h. And this induction was for seven of nine analyzed genes (the exceptions were *GGT* and *IDH*) significantly different from the controls. Interestingly, after short-term exposure for 2 h, the genes responded with a slight intensification of transcription, and, for some genes, it was significantly different from the corresponding controls (*GSS*, *GR*, *GPX*, *GSTT1*, *GSTa* and *GSTL3*). However, the treatment lasting 4 h exhibited the lowest values for all analyzed gene expression, with the exception of *GSTL3*. For *IDH*, it was even decreased compared to the control (log_2_FC = −3.3).

**Figure 8:**
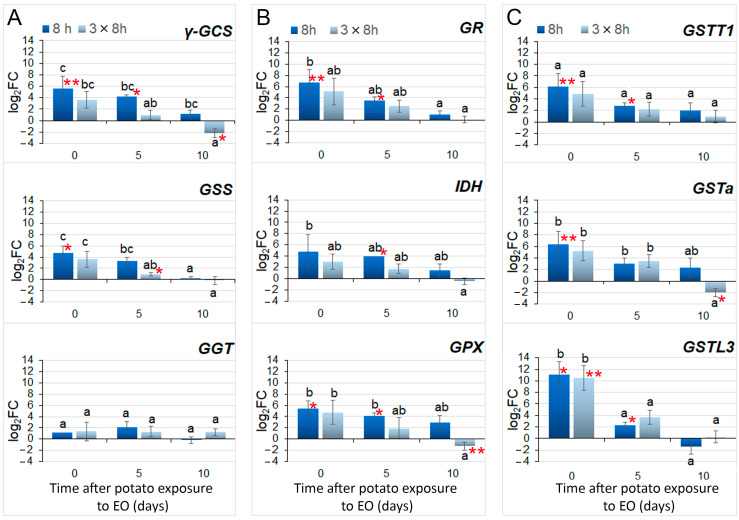
RT-qPCR obtained expression profiles of potato genes involved in GSH metabolism. Expression of genes involved in the (**A**) biosynthesis of GSH, (**B**) the redox conversion of GSH, and (**C**) conjugating GSTs was examined in plants immediately (0 days) after exposure to French marigold EO for single (8 h) or three-times-repeated 8 h long (3 × 8 h) periods, or after 5 and 10 days. The fold change (FC) of the gene’s expression was obtained after ΔΔCt normalization to the expression of reference 18S gene and to the expression in unexposed controls (log_2_FC = 0) for each time point, and presented after log_2_ transformation (log_2_FC). Means of three biological replicates (n = 3) were compared with one-way ANOVA and presented with standard errors. The letters above the bars denote significant differences according to Fisher’s LSD post-hoc test at *p* ≤ 0.05. For each gene at each time point, a Student’s *t*-test was used for comparison between treatment and corresponding control, and statistically different means are denoted with one asterisk (*) for *p* ≤ 0.05 or with two asterisks (**) for *p* ≤ 0.10.

When the expression patterns of GSH metabolism-related genes were analyzed in potato plants exposed to single (8 h) or repeated (3 × 8 h) FM-EO treatment just upon exposure, or 5 and 10 days upon exposure (Figure 1C), the results of the RT-qPCR showed a decline in expression over time (Figure 8). For almost all genes belonging to each of three analyzed gene groups (GSH biosynthesis and degradation (Figure 8A), the GSH-GSSG redox cycle (Figure 8B) and GSTs glutathionylation (Figure 8C)), repeated exposure (light blue bars) slightly decreased the accumulation of transcripts compared to single exposure (dark blue bars), but this alternation was not significantly different (with one exception of *ƴ-GCS* after 10 d). The decreasing expression trend was generally persisted over time after the exposure (either 8 h or 3 × 8 h), with expression levels returning to those recorded for controls mostly after 10 days. The initial increased expression (after 8 h of FM-EO exposure), recognized by *t*-tests between treatments and controls, remained elevated in exposed plants after 5 days of exposure for all genes except *GGT* and *GSTa*. Of all the analyzed genes, only *GGT* did not show any changes in expression, affected neither by repeated exposure nor the passage of time. Interestingly, for some of the genes (*ƴ-GCS*, *GPX* and *GSTa*), repeated exposure caused further decrease in expression after 10 days, whit expression levels even lower than in controls.

## 4. Discussion

Many of the atmosphere’s volatile organic compounds (VOCs) are of natural origin, with most biogenic VOC emissions originating from vegetation. Compared to anthropogenic sources, biogenic VOCs have a shorter lifetime in the troposphere, as they react intensively with the highly reactive hydroxyl radicals (^•^OH). After emission, VOCs withstand ^•^OH-induced oxidation, which leads to the formation of ozone and secondary organic aerosol particles or deposition on the surface [41]. VOCs can adhere to the surfaces of a recipient plant without the need for further modification, or the recipient plant can respond to VOC through induced molecular or physiological changes [42,43,44]. More recently, it has been proposed that VOCs could be considered damage-associated molecular patterns (DAMPs), alarm signaling molecules [45,46] that can trigger direct and indirect defense in distant parts of the same plant or neighboring plants [47,48]. Upon reception of the volatile signals, there is a strong depolarization of the plasma membrane of the recipient plant cells [49] and an induction of ROS production [50]. The results obtained in this study confirmed that the perceived volatiles induce changes in the oxidative state of the receiving potato plants without causing an outbreak. Different types of ROS are produced in plants, and electron paramagnetic resonance (EPR) spectroscopy, which provides positive signals only in the presence of free radicals, was used to detect radicals in the samples. However, since hydroxyl radicals are very reactive and have a lifetime of about 10 ns [51], we used a spin trap that generates longer-lived radical species that are stable for minutes or even hours when reacting with very reactive radicals [52]. DEPMPO is a spin trap that reacts with various short-lived radicals, and with ^•^OH it forms DEPMPO/OH adducts, which have a spectrum with a specific hyperfine structure [53]. According to the results, the exposure of potato plants to FM-EO promoted the accumulation of hydroxyl radicals during the first hours. However, after the initial increase, the accumulation of ^•^OH decreased compared to the controls. After a detailed analysis of the experiments on kinetics and ^•^OH accumulation, it became clear that the closing of the jars triggered ROS accumulation in the unexposed control plants. The duration of the experiment correlated directly with the extent of these circumstances. However, prolonged exposure (8 h) to the environment saturated with FM-EO volatiles caused the exposed plants to adapt via a reduced accumulation of radicals or the activation of the antioxidant defense system and the successful mitigation of EO-induced oxidative alternation. The reduced ^•^OH accumulation and a primed physiological adaptation ultimately contributed to plant survival under stress conditions.

The subsequent analysis of the radical scavenging activity of the potato plants showed that the exposure of the potato plants to FM-EO neither leads to a significant radical scavenging activity towards DPPH radicals, nor does it change significantly with the exposure interval. Already after 2 h of closing the jars with control plants, the scavenging activity towards DPPH radicals was induced, and it remained higher in the control plants than in the exposed plants, indicating that prolonged exposure to FM-EO does not induce such a strong stress signal that could cause a strong antioxidant response leading to a disturbed plant homeostasis. These results are consistent with the results of the kinetics of the reduction of 3CP and 3CxP spin probes. However, the exact opposite trend was observed for scavenging activity towards the physiologically more relevant ascorbyl radical (^•^Asc), with exposed plants exhibiting higher long-term scavenging activity. Ascorbate is a water-soluble cellular reducing agent with properties that make it a superior donor antioxidant in biological systems [54]. Its one-electron oxidation generates the EPR-detectable ascorbyl radical. The detection of ascorbyl radicals by EPR is easy due to their long lifetime in contrast to hydroxyl radicals. Due to the resonance stabilization of the unpaired electron, it is a relatively unreactive free radical [55].

ROS are paramagnetic species which, as already mentioned, can be detected by EPR. However, since ROS in plants are very reactive and short-lived, it is difficult to detect them with EPR. On the other hand, the spin-trap technique has some disadvantages, such as the application of high spin-trap concentrations and possible toxic effects that may occur [56]. Therefore, the reducing capacity and the presence of oxidative stress in plants is determined using pyrrolidine spin probes, which are suitable for ROS detection in plants and react very quickly [36]. The probes react with ROS and form relatively stable products [57]. The spin probes used in this study, 3CP and 3CxP, belong to a class of stable pyrrolidines with long half-lives. Considering that 3CP is lipophilic and membrane permeable and simultaneously measures intracellular and extracellular radicals, while the hydrophilic 3CxP is membrane impermeable and allows for the measurement of extracellular redox status, the combination of these probes enables the kinetic monitoring of physiological radicals. Although the use of spin probes does not specify the radical species, it provides information on the redox status and localization of free radical generation. Pyrrolidine spin probes in plant cells can be reduced by glutathione-related enzymes and provide information about the antioxidant status of the plant [58]. The shape of the curves shows that the extracellular activity changed only minimally after 2 h, while the reduction in the probe intracellularly was slower, indicating the onset of a stress response in the plant. This trend persisted and became even more pronounced in plants exposed to FM-EO for 8 h. This intracellular effect is expected to gradually extend to the extracellular space after prolonged exposure, indicating a systemic response to oxidative stress. Interestingly, in potato plants exposed to FM-EO for 8 h, the probe signal reaches a plateau after an initial rapid reduction of 3CP intracellularly, indicating that the antioxidant system acts lethargically and/or that the probe has been reoxidized by ROS.

After an 8 h exposure to FM volatiles, the potato plants showed changes in the expression of genes involved in glutathione metabolism. Of the total number of glutathione genes, 35% showed altered expression, of which 97.7% were upregulated [29]. These genes belong to the glutathione redox metabolic pathways, but the highest expression level was found in the GSTs gene group involved in the glutathionylation process. There is ample evidence that GST is responsible for the detoxification of xenobiotics and the removal of ROS caused by herbicides, pathogenic attacks or air pollutants [59]. The glutathione S-conjugates obtained are then transported into the vacuole and further metabolized [60]. GST also plays an irreplaceable role in the transmission of defense signals [16,61]. According to our results, the exposure of potato plants to FM-EO triggered the oxidation–reduction cycles of glutathione with an increased expression of GSTs, responsible for metabolic changes in the perceived exogenous volatiles. The RT-qPCR results confirmed the microarray results regarding the altered expression of genes involved in the glutathione redox cycle and the high expression of GST after 8 h of exposure to FM-EO. It is clearly known that GSTs are induced by oxidative stress caused by various stress stimuli, but in some cases GST induction is specific to the specific stressor [62,63]. This can be explained by the fact that the promoters of the GST gene have different regulatory elements that respond differently to specific or more general stress-induced signals [64,65]. It has already been shown that GST regulation can be altered by agrochemicals, including herbicide safeners and synergists [66,67]. In addition, many plant–pathogen interactions have been associated with significant accumulation of numerous GST transcripts and proteins, as well as higher overall GST enzyme activity.

The results of total glutathione showed a significant decrease in total glutathione content in FM-EO-exposed plants after the first 2 h, while the glutathione level reached control levels after 8 h. This observation is consistent with the increasing expression of genes involved in glutathione metabolism during the 8 h of exposure to FM-EO. However, the initial reduction could also be a consequence of the intense conjugation of glutathione with VOCs and their successful cellular sequestration. As expected, the reduced form of glutathione dominated in all samples, both in the EO-exposed or unexposed plants. However, after the first 2 h, GSSG increased, as a result of induced ROS formation and glutathione activity towards accumulated ^•^OH. At this time, the VOCs accumulated to such an extent that they enhanced the oxidation of GSH in the exposed potato plants. After an 8 h exposure to FM-EO, the level of oxidized GSSG form was back to the control level, which correlates with an increased expression of genes involved in glutathione biosynthesis and redox cycling, replenishing the reserves of this antioxidant.

Driven by the idea that essential oil could induce a physiological state in which exposed potato plants are able to activate the defense response faster and thus act as a priming agent, repeated exposure and its sustained effect were evaluated. Available data showed that a second signaling event can rapidly activate the accumulated signaling proteins in primed plants and enhance the defense response, contributing to an intergenerational stress memory in plants [68,69]. Repeated exposure to FM-EO did not induce new peaks in the expression of the studied potato genes involved in GSH metabolism. On the contrary, all studied genes showed reduced expression in plants repeatedly exposed to FM-EO compared to plants exposed once, indicating that the potato plants recognize and adopt to the initial volatile signal. This conclusion is imposed even after the analysis of total glutathione after repeated exposure to FM-EO. These results showed a slightly increased glutathione content compared to the control, but were comparable with glutathione content after single 8 h exposure. Glutathione remained at a similar level as in the control potato plants 5 or 10 days after the end of exposure. Repeated exposure discretely decreased the total glutathione and the proportion of its oxidized form in the exposed potato plants compared to single exposure, and the content remained constant until the end of the experiment. This means that the repeated exposure did not affect the recipient potato plants by an intense change in glutathione, suggesting the absence of stress and adaptation/tolerance to the volatile cue of FM-EO, and that more intense changes in glutathione content occur during the first hours of exposure to the essential oil.

The molecular mechanisms behind VOCs-induced resistance are still poorly understood, with an epigenetic reprogramming, such as DNA methylation, considered responsible for the fine-tuning of plant resistance. The available data had shown that the primed state is related to the enhanced perception of stress signals, rather than to direct gene induction, utilizing minimal plant fitness costs [13,70]. Epigenetic changes make gene promoters more accessible and easier to activate, supporting the phrase “priming smells like epigenetics”.

## 5. Conclusions

**Figure 9:**
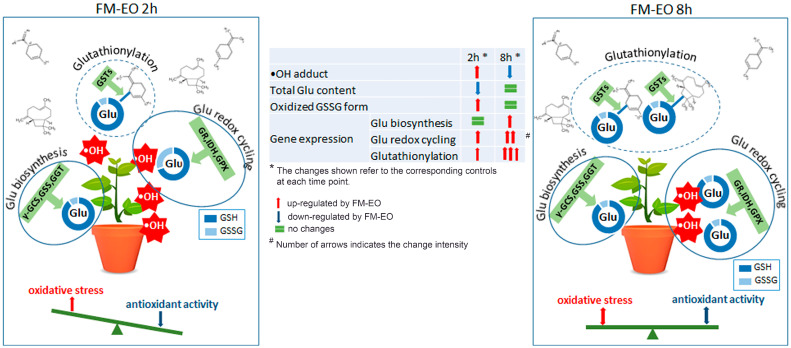
Schematic model summarizing the most significant results obtained when studying potato plants’ early responses to French marigold essential oil (FM-EO) within 2 and 8 h. The measurement of the presence of ^•^OH adducts, total glutathione (Glu) content, the ratio between reduced (GSH) and oxidized (GSSG) forms and the expression of genes involved in Glu biosynthesis, Glu redox cycle and glutathionylation showed the relationship between FM-EO-induced oxidative stress and Glu-related antioxidant response.

Using state-of-the-art analytical methods and a comprehensive experimental design, we confirmed the perception of VOCs in the potato plants and proved the involvement of glutathione in the initial response (Figure 9).

Already after 2 h of exposure to FM-EO, the high yield of ^•^OH radicals indicated an altered physiology of the potato plants. An increased proportion of the oxidized form of glutathione indicated an active involvement of this antioxidant in scavenging the EO-induced accumulation of free radicals’. At the same time, a significant decrease in total glutathione indicated the intense conjugation and cellular sequestration of VOCs. However, after 8 h of exposure, when a significant increase in the expression of glutathione-related genes occurred, the level of ^•^OH radicals decreased sharply, most likely due to intensive removal by glutathione redox cycling. An unchanged total glutathione level and a stabile predominance of the reduced over the oxidized form, led to a newly established equilibrium toward the adaptation to interspecies communication. However, the involvement of enzymatic antioxidants, such as ascorbate peroxidase, dehydroascorbate reductase and glutathione reductase, should not be disregarded and will be part of our future study. The intense accumulation of GST transcripts throughout the period of EO exposure suggests the glutathionylation process is involved in this adaptation. This emerges as a promising defense strategy in which VOCs, stored in vacuoles, can be used in a future pest attack. This pathway will be the focus of our future study, with the aim of unveiling this mechanism in detail and using the knowledge gained to propose green approaches to improving the sustainability of agricultural production. Finally, we can conclude that glutathione metabolism was affected by French marigold VOCs in receiving potato plants, and that glutathione molecules were involved in the mitigation of VOC-induced oxidative burst during the early responses.

## Data Availability

The data supporting the findings of this study are available at the RADaR—Digital Repository (http://radar.ibiss.bg.ac.rs/handle/123456789/7014) or on reasonable request from the corresponding author.

## Supplementary Materials

The following supporting information can be downloaded at https://www.mdpi.com/article/10.3390/antiox13121565/s1, Table S1: Sequences of primers used in RT-qPCR analyses.
