# Supplementary material for: Glutathione Involvement in Potato Response to French Marigold Volatile Organic Compounds
Source: Antioxidants (Basel). 2024 Dec 19;13(12):1565. doi: 10.3390/antiox13121565 (PMC11673417; doi:10.3390/antiox13121565)
Supplement: Supplementary file 1 [file antioxidants-13-01565-s001.zip › antioxidants-3256885-supplementary.pdf]

Table S1. Sequences of primers used in RT-qPCR analyses

Gene	GeneBank Acc No	enzyme name	F primer (5'-3')	R primer (5'-3')	Amplicon size (bp)
γ-GCS	XM_006343925.2	glutamate--cysteine ligase	TCTGGAAATGAGGGGTGCTG	TTCCGCAGTCCAATCAGACG	133
GSS	XM_006361818.2	glutamate--glutathione synthetase	TTGTCTGGTCAGCGAGCTTC	ATCACAGCCCTTGGATCACC	156
GGT	XM_006353446.2	gamma-glutamyltranspeptidase 3	GGTGGCGGTTTTATGGTTGT	TCGACCAAGCTGTGTGAAGA	184
GR	XM_006360297.2	glutathione reductase	CGTGCTGTGATACTTGGTGG	TCGTGCAAGGATGCATAGTGA	184
GPX	XM_006343933.2	probable phospholipid hydroperoxide glutathione peroxidase	TGTCAAGGATGCTAAGGGCA	AGGGAAGGCCAGAATCTCCA	169
IDH	XM_006354059.2	isocitrate dehydrogenase [NADP]	CCAAGTACGAGGAAGCTGGAA	CAGGACACACCAGGACAGAA	186
GSTL3	XM_006338992.2	glutathione S-transferase L3-like	CACATCTGGGAGGCCAAAAC	GCACGAAGCACCCCTTATTC	185
GSTT1	XM_006360625.2	glutathione S-transferase T1-like	GGTCAACATCGCACTCCTGA	TGGTGCCAATCCAAGACACA	200
GSTa	XM_006355737.2	glutathione S-transferase	ATCCATTTGGTCAAGTTCCAGC	GGGGTCGAATTTCTGGGCT	182
